# Ischemic vs. Nonischemic Cardiomyopathy in TAVR for Moderate Aortic Stenosis: A TAVR UNLOAD Sub Analysis

**DOI:** 10.1016/j.shj.2025.100787

**Published:** 2025-12-19

**Authors:** Philipp von Stein, Henning Guthoff, Björn Redfors, Julia B. Thompson, Ernest Spitzer, Philippe Pibarot, Jeroen J. Bax, Jan G.P. Tijssen, Michael L. Chuang, Yukari Kobayashi, Arsalan Abu-Much, Nicole Cristell, Alexandra Popma, David J. Cohen, Sammy Elmariah, Martin B. Leon, Nicolas M. Van Mieghem, the TAVR UNLOAD trial study investigators

**Affiliations:** a Cardiovascular Research Foundation, New York, New York, USA; b Department of Cardiology, Heart Center, Faculty of Medicine, University of Cologne, Cologne, Germany; c Center for Cardiovascular Medicine ABCD, Aachen – Bonn – Cologne – Düsseldorf, Cologne, Germany; d Department of Population Health Sciences, Weill Cornell Medicine, New York, New York, USA; e Department of Molecular and Clinical Medicine, Gothenburg University, Gothenburg, Sweden; f Department of Cardiology, Sahlgrenska University Hospital, Gothenburg, Sweden; g Department of Cardiology, Thoraxcenter, Cardiovascular Institute, Erasmus University Medical Center, Rotterdam, The Netherlands; h Cardialysis, Rotterdam, The Netherlands; i Department of Medicine, Québec Heart and Lung Institute, Laval University, Québec City, Québec, Canada; j Department of Cardiology, Leiden University Medical Center, Leiden, The Netherlands; k Academic Medical Center, University of Amsterdam, Amsterdam, The Netherlands; l Columbia University Irving Medical Center/New York-Presbyterian Hospital, New York, New York, USA; m St. Francis Hospital and Heart Center, Roslyn, New York, USA; n Division of Cardiology, Department of Medicine, University of San Francisco, San Francisco, California, USA

**Keywords:** Aortic stenosis, AS, cardiomyopathy, ICM, Ischemic, Moderate, TAVR, Transcatheter aortic valve replacement

## Abstract

• This exploratory analysis of the Transcatheter Aortic Valve Replacement to UNload the Left ventricle in patients with ADvanced heart failure trial did not identify evidence of a differential treatment effect of transcatheter aortic valve replacement compared to clinical aortic stenosis surveillance according to cardiomyopathy etiology (ischemic versus nonischemic) in patients with heart failure with reduced ejection fraction and moderate aortic stenosis.
• The study was underpowered to definitively exclude a potential modifying effect of cardiomyopathy etiology, and any differential treatment response may require longer follow-up to become apparent.

Transcatheter aortic valve replacement (TAVR) has transformed the management of severe aortic stenosis (AS), but its role in moderate AS remains unclear—particularly in patients with heart failure (HF) with reduced ejection fraction (HFrEF), who experience high rates of morbidity and mortality.^1^ The international, randomized TAVR UNLOAD trial (Transcatheter Aortic Valve Replacement to UNload the Left ventricle in patients with ADvanced heart failure; NCT02661451) reported that transfemoral TAVR was not superior to clinical aortic stenosis surveillance (CASS) in improving outcomes in patients with symptomatic HFrEF (resting left ventricular ejection fraction [LVEF] <50%) and moderate AS.^2^ However, the high prevalence of ischemic cardiomyopathy (ICM) in the study may have attenuated the overall treatment effect.^2^ Whether outcomes differ based on cardiomyopathy etiology (ICM vs. non-ICM [NICM]), has not previously been investigated and is the focus of this sub analysis.

ICM was defined by the site-reported presence of coronary artery disease, prior myocardial infarction, prior percutaneous coronary intervention, or prior coronary artery bypass grafting; NICM by the absence of all of these. We evaluated the 1-year composite of all-cause death or HF hospitalization and its individual components, stratified by cardiomyopathy etiology. Changes in LVEF and Kansas City Cardiomyopathy Questionnaire Overall Summary Score (KCCQ-OSS) from baseline to 1 year were analyzed using baseline-adjusted models that included the respective baseline value of each endpoint as the sole covariate. Treatment-by-etiology interaction terms tested heterogeneity of effect for each endpoint.

Between January 2017 and December 2022, 178 participants (age 77.4 ± 7.2 years; 20.8% females; 55.6% New York Heart Association class III/IV; LVEF 37.8% ± 6.9% [after correction of previous inconsistencies in LVEF assessment], KCCQ-OSS 55.8 ± 23.1) were 1:1 randomized. Of these, 143 patients (80.3%) had ICM and 35 (19.7%) had NICM. Cardiomyopathy etiology was balanced between treatment groups (ICM: 78.7% in TAVR vs. 82.0% in CASS; NICM: 21.3% in TAVR vs. 18.0% in CASS). ICM patients were more often male (83.9 vs. 60.0%) and more frequently had arterial hypertension (86.7 vs. 64.7%), hyperlipidemia (73.4 vs. 48.6%), and carotid artery disease (21.1 vs. 0.0%, all *p* ≤ 0.008), with otherwise balanced clinical baseline characteristics. One ICM patient randomized to TAVR underwent percutaneous coronary intervention concomitantly with TAVR.

The 1-year composite endpoint showed a numerical trend favoring TAVR over CASS in both ICM and NICM. However, none of these differences reached statistical significance, and no treatment-by-etiology interaction was observed (p_interaction_ = 0.853; Figure 1a). Similar patterns were observed for the individual components of the composite endpoint, without statistically significant differences across etiologies (Figure 1b). LVEF increased in both treatment arms across etiologies. KCCQ-OSS improved with TAVR in both etiologies, whereas changes with CASS were not significant. No treatment-by-etiology interaction was observed for either LVEF change or KCCQ-OSS change (Figure 1c).

**Figure 1:**
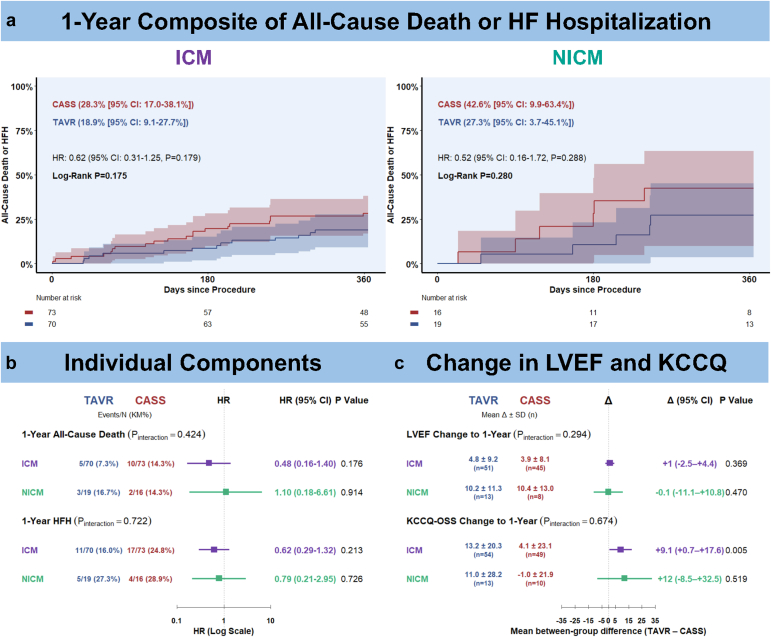
**One-year outcomes by cardiomyopathy etiology.** (a) Kaplan–Meier curves for the 1-year composite of all-cause death or HF hospitalization in ICM (left) and NICM (right). Among patients with ICM, 13 of 70 treated with TAVR and 20 of 73 treated with CASS experienced the composite endpoint within 1 year. Among those with NICM, 5 of 19 undergoing TAVR and 6 of 16 undergoing CASS reached the composite endpoint. In NICM, the early numerical separation should be interpreted with caution given the low number of patients and events and the resulting wide CI. (b) Forest plot summarizing 1-year all-cause death and HF hospitalization. HR < 1 favors TAVR. (c) Baseline-to-1-year changes in LVEF and KCCQ-OSS by etiology. Only paired cases are included, and columns show mean absolute changes. Plotted mean differences are unadjusted; row and interaction *p* values are baseline adjusted. Positive differences favor TAVR. Abbreviations: CASS, clinical aortic stenosis surveillance; CI, confidence interval; ICM, ischemic cardiomyopathy; HF, heart failure; HFH, heart failure related hospitalization; HR, hazard ratio; KCCQ-OSS, Kansas City Cardiomyopathy Questionnaire Overall Summary Score; LVEF, left ventricular ejection fraction; NICM, nonischemic cardiomyopathy; TAVR, transcatheter aortic valve replacement.

In summary, this exploratory analysis of the TAVR UNLOAD trial did not identify evidence of a differential treatment effect of TAVR compared to CASS according to cardiomyopathy etiology in patients with HFrEF and moderate AS. Although numerical differences were observed, these findings were not statistically significant and were particularly limited by the small number of NICM patients. Accordingly, these results must be interpreted in light of the overall neutral trial and the relatively small number of events within subgroups. The study was underpowered to definitively exclude a potential modifying effect of cardiomyopathy etiology, and any differential treatment response may require longer follow-up to become apparent. Mechanistically, unloading of the left ventricle may alleviate afterload mismatch and promote reverse remodeling in NICM, whereas in ICM, benefits may stem from increased transvalvular flow and improved coronary perfusion, particularly in the presence of hibernating myocardium. Known differential responses to afterload reduction and medical HF therapies further highlight the importance of considering HFrEF etiology in future research.^3^^,^^4^ Additional insights into the role of TAVR in moderate AS are expected from the ongoing PROGRESS (NCT04889872) and Evolut EXPAND TAVR II (NCT05149755) trials.

## Ethics Statement

This study was conducted in accordance with the Declaration of Helsinki and all applicable regulatory requirements. The study protocol and all amendments were approved by the responsible Institutional Review Boards or Ethics Committees at each participating center. All patients provided written informed consent before enrollment.

## Funding

The TAVR UNLOAD trial was supported by Edwards Lifesciences.

## Disclosure Statement

Björn Redfors reports consultant fees from 10.13039/100004319Pfizer and 10.13039/100001003Boehringer Ingelheim. Ernest Spitzer reports institutional contracts/grants for which he receives no direct compensation from 10.13039/100000046Abbott, Biosensors Europe SA, 10.13039/100008497Boston Scientific, 10.13039/100006520Edwards Lifesciences, 10.13039/100004374Medtronic, Mixin Medtech (Suzhou) Co., Ltd., Shanghai Microport Medical Co., Ltd., NVT GmbH, Philips Healthcare, Pie Medical Imaging, Shanghai Shenqi Medical Technologies Co., Ltd., and Siemens Healthcare GmbH. Philippe Pibarot is the Canada Research Chair in Valvular Heart Disease; his research program is funded by the 10.13039/501100000024Canadian Institutes of Health Research (grant FDN-143225), Ottawa, Ontario, Canada; has received funding from 10.13039/100006520Edwards Lifesciences, 10.13039/100004374Medtronic, Pi-Cardia, and Cardiac Phoenix for echocardiography core laboratory analyses and research studies in the field of transcatheter valve therapies, for which he received no personal compensation; and has received lecture fees from 10.13039/100006520Edwards Lifesciences and 10.13039/100004374Medtronic. Jeroen J. Bax reports the Department of Cardiology (LUMC, The Netherlands) has received research grants from 10.13039/100004374Medtronic, Biotronik, 10.13039/100006520Edwards Lifesciences, and 10.13039/100008497Boston Scientific; and he has received speaker fees from Abbott Vascular. Alexandra Popma reports that her spouse is a former employee of and holds non-vested equity in 10.13039/100004374Medtronic. David J. Cohen reports institutional research support from 10.13039/100000046Abbott, 10.13039/100006520Edwards Lifesciences, 10.13039/100008497Boston Scientific, Philips, Corvia Medical, ZOLL, CathWorks, and ANCORA and consulting income from 10.13039/100004374Medtronic, 10.13039/100006520Edwards Lifesciences, 10.13039/100000046Abbott, 10.13039/100008497Boston Scientific, and Elixir Medical. Sammy Elmariah reports institutional research support from 10.13039/100006520Edwards Lifesciences, 10.13039/100004374Medtronic, and 10.13039/100000046Abbott; consulting fees from 10.13039/100006520Edwards Lifesciences; and equity in Prospect Health. Martin B. Leon reports institutional research support from 10.13039/100006520Edwards Lifesciences, 10.13039/100004374Medtronic, 10.13039/100008497Boston Scientific, and 10.13039/100000046Abbott; and consulting/advisory board participation for Foldax, Anteris, JenaValve, Medinol, SoloPace, and Bain Capital. Nicolas M. Van Mieghem has received grant support from Abbott Vascular, 10.13039/100008497Boston Scientific, 10.13039/100006520Edwards Lifesciences, and 10.13039/100004374Medtronic; and advisory fees from 10.13039/100000046Abbott, 10.13039/100008497Boston Scientific, Pulse Cath BV, and 10.13039/100004374Medtronic. The other authors report no potential conflicts of interest.
